# Differences of Intrasession Reproducibility of Circumpapillary Total Retinal Thickness and Circumpapillary Retinal Nerve Fiber Layer Thickness Measurements Made with the RS-3000 Optical Coherence Tomograph

**DOI:** 10.1371/journal.pone.0144721

**Published:** 2015-12-14

**Authors:** Yoshiyuki Kita, Gábor Hollό, Ritsuko Kita, Daisuke Horie, Makoto Inoue, Akito Hirakata

**Affiliations:** 1 Department of Ophthalmology, Kyorin University School of Medicine, Tokyo, Japan; 2 Department of Ophthalmology, Semmelweis University, Budapest, Hungary; Casey Eye Institute, UNITED STATES

## Abstract

**Purpose:**

To evaluate the intrasession reproducibility of various thickness parameters used to diagnose and follow-up glaucoma, in particular circumpapillary total retinal thickness (cpTR) provided by the RS-3000 optical coherence tomograph (OCT).

**Methods:**

Fifty-three healthy eyes of 28 subjects underwent three consecutive imaging with the RS-3000 Advance OCT (NIDEK, Aichi,Japan) to evaluate the intrasession reproducibility of circumpapillary total retinal thickness (cpTR), circumpapillary retinal nerve fiber layer thickness (cpRNFL), macular ganglion cell complex thickness (mGCC) and macular total retina thickness (mTR) measurements. Intraclass correlation (ICC), coefficient of variation (CV) and reproducibility coefficient (RC) were calculated for each parameter.

**Results:**

The ICC and CV values for mean cpTR and cpRNFL were 0.987 and 0.897, and 0.60% and 2.81%, respectively. The RC values for the mean cpTR and cpRNFL were 5.95 μm and 9.04 μm, respectively. For all cpTR parameters the ICC values were higher and both the CV and RC values were lower than those for the corresponding cpRNFL parameters. The ICC and CV values for superior mGCC, inferior mGCC, superior mTR and inferior mTR were 0.983, 0.980, 0.983 and 0.988, and 0.84%, 0.98%, 0.48% and 0.43%, respectively. The RC values for superior mGCC, inferior mGCC, superior mTR and inferior mTR were 2.86 μm, 3.12 μm, 4.41μm and 4.43 μm, respectively.

**Conclusions:**

Intrasession reproducibility of cpTR, mGCC and mTR measurements made on healthy eyes was high. Repeatability of cpTR measurements was better than that of the corresponding cpRNFL measurements. These results suggest that future clinical investigations addressing detection of glaucoma and glaucomatous progression with the RS-3000 OCT may benefit from focusing on the cpTR parameters.

## Introduction

In the last decade spectral domain optical coherence tomography (SD-OCT) gained an increasing clinical importance in detecting glaucoma and glaucomatous structural progression during long term disease management [1–7]. The several different SD-OCTs have different technical features, including illumination wavelength, segmentation algorithm, size of the image area, normal database, measurement precision and reproducibility [1–9]. Due to the above between-instrument differences determination of parameters performing best for glaucoma follow-up needs to be made for each SD-OCT instrument, respectively. For several SD-OCT instruments, which have been in clinical use for many years, this assessment has been made [10–13], but for some more recently introduced systems the evaluation is still needed.

The RS-3000 Advance (NIDEK, Aichi, Japan) is a relatively new SD-OCT instrument [5, 9]. Its wavelengths is 880 nm, and the instrument acquires 53,000 A scans per second with an axial resolution of 7 μm, a lateral resolution of 20 μm, and a scan depth of 2.1 mm. The current software of the RS-3000 OCT (software version 1.4.2.1) offers a unique function for follow-up image capture, in order to increase the measurement reproducibility [14]. Image alignment is based on scanning laser ophthalmoscope (SLO) images obtained immediately prior to image capture. This eliminates the relatively long image acquisition time, which is necessary to image capture when the automatic tracking function is used. The SLO based image alignment function is expected to considerably reduce motion artefacts via pre-acquisition tracking. The retinal vessels are automatically identified on the SLO images, and are used for correct positioning of the subsequent OCT scan. When the baseline and the subsequent SLO images are perfectly aligned, the actual OCT scan is automatically acquired without active tracking of eye movements during the acquisition procedure [15]. The software of the RS-3000automatically offers a protocol to measure various circumpapillary total retinal (cpTR) and macular total retinal (mTR) thickness parameters in addition to providing the corresponding circumpapillary retinal nerve fiber (cpRNFL) and macular ganglion cell complex (mGCC) thickness parameters [15].

Conventional SD-OCTs do not automatically provide data on cpTR thickness. This is probably due to the current clinical practice in which use of cpTR using is not a part of the routine. Till now only one investigation on cpTR thickness measurements made with an SD-OCT system was reported [16].

The purpose of our current study was to evaluate and compare the intraobserver intrasession reproducibility (repeatability) of the various corresponding cpTR and cpRNFL thickness, and mGCC and mTR thickness parameters, respectively, using the SLO based image alignment function in normal eyes. We wished to evaluate the usefulness of the SLO-based image alignment technique, and to identify parameters with potential clinical usefulness for future glaucoma diagnostic and follow-up studies.

## Subjects and Methods

This prospective cross sectional study was made on healthy Japanese participants between February 2015 and April 2015 at the Kyorin Eye Center in Tokyo, Japan. The Institutional Review Board for Human Research at the Kyorin University approved this study. The corresponding consent form followed the tenets of the Declaration of Helsinki. Informed consent was obtained from all participants before enrolment and written informed consent was obtained from each participants. All applicable institutional regulations concerning the ethical use of human volunteers were followed during this research.

All participants underwent a complete ophthalmologic examination including medical and family history; visual acuity testing (including refraction); slit-lamp biomicroscopy; gonioscopy; Goldmann applanation tonometry and stereoscopic fundus examination via a dilated pupil. The visual field (VF) was investigated with the 24–2 Swedish Interactive Threshold Algorithm test of the Humphrey Field Analyser (Humphrey-Zeiss Systems, Dublin, CA). The visual fields were considered reliable if fixation losses were <20% and both false-positive and false-negative rates were <15%. Axial length was measured with a light interferometry (OA-1000, TOMEY Corporation, Aichi, Japan). Non-cycloplegic refraction was determined using an auto ref/keratometer (ARK-530; NIDEK, Aichi, Japan). Refraction data were converted to spherical equivalents (SE), which were calculated by the additions of spherical refractive error values (in dioptres [D]) to one-half of the cylindrical refractive power. Participants with a history of intraocular surgery or ophthalmic laser procedures were not included. Furthermore, no subject with a possible history of elevated intraocular pressure (IOP) (e.g. iridocyclitis, ocular trauma), other intraocular eye disease, family history of glaucoma in a first degree relative, diabetes, or any other diseases that could affect the result of the visual field testing and OCT examination (e.g. pituitary lesions, demyelinating disease) was included. Participants were required to have an IOP of <21 mm Hg, a normal optic nerve head appearance, normal open anterior chamber angles, normal VF test results, a best-corrected decimal visual acuity of ≥1.0, a spherical equivalent between +1.00 and −12.00 D and a cylindrical refractive error of ≤3.00 D. The optic nerve head appearance was considered normal if all of the following criteria were met: symmetrical vertical cup to disc (C/D) ratio of <0.7; uniform neuroretinal rim and no clinically visible RNFL defects or optic nerve changes (e.g. diffuse or localised rim thinning, disc haemorrhage, vertical C/D ratio >0.2 different from the fellow eye). Participants with myopic macular degeneration, posterior staphyloma, tilted discs, or peripapillary atrophy extending to the measurement circle of the OCT were not included in the current investigation.

### Optical coherence tomography

The OCT measurements were made with an RS-3000 Advance OCT (NIDEK, Aichi, Japan; software version 1.4.2.1) [5, 9]. The RS-3000 instrument includes a confocal scanning laser ophthalmoscope allowing fundus images to be monitored and SD-OCT equipment allowing 3D tomographic imaging. RS-3000 collects ocular microstructural images using a scanning laser diode that emits a scan beam with a wavelength of 880 nm. The OCT equipment has a 7-μm tissue depth resolution and 20 μm transverse resolution. Single 3D data sets are acquired in 1.6 seconds. Automated measurement of various cpRNFL, cpTR, mGCC and macular total retinal (mTR) thickness parameters is provided by the built-in software. A follow-up image capture function of the RS-3000 OCT offers image alignment prior to image capture. This function is intended to increase reproducibility of the image capture procedure [14]. The image capture function uses retinal vessels automatically identified on the baseline SLO image to correctly position the subsequent scan. After the two SLO images are perfectly aligned, the new scan is automatically acquired without active tracking of eye movements during the acquisition procedure. In the current investigation this function was applied, and pupil dilation was not performed. Three high-quality images were obtained by the same skilled operator (Y. K.). Only scans with a Signal Strength Index (SSI) higher than 7 were used in analyses. The 3 OCT scans were acquired with approximately 5 minutes intervals. The participants were required to remove their chin from the chinrest between the image acquisition sessions.

#### Circumpapillary OCT parameters

**Fig 1:**
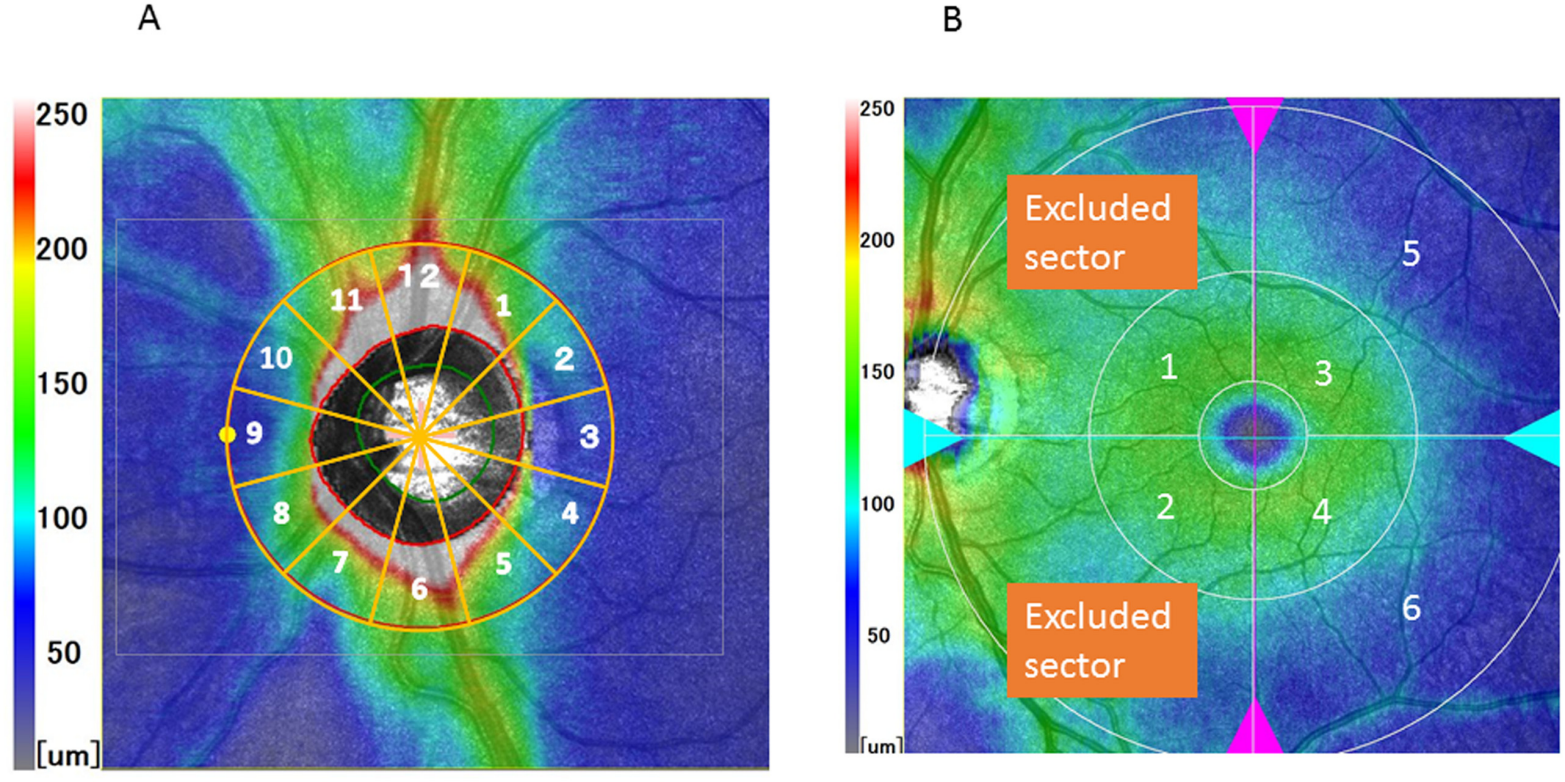
Sectors of the circumpapillary area (A) and the macula area (B) used for analysis in the current investigation. The nasal part of the outer ring at 180 degrees within macula area was excluded from the analysis.

For cpRNFL and cpTR imaging, raster scanning over a 6 × 6 mm^2^ area centered on the optic disc center was conducted at a scan density of 512 A-scans (horizontal) × 128 B-scans (vertical) [5]. Measurements of cpRNFL and cpTR thicknesses were performed using a 3.45-mm-diameter circle automatically positioned around the optic disc in each 3D data set. The cpTR thickness was measured between the internal limiting membrane (ILM) and the outer retinal pigment epithelium (RPE). The following software parameters were used to evaluate cpRNFL and cpTR thickness: (1) average thickness of the entire 360° around the optic nerve head; (2) superior quadrant; (3) inferior quadrant; (4) temporal quadrant; (5) nasal quadrant and (6) all 12 separate sectors. For reference purposes, sectors of equal size (30.0°) were numbered in sequence from the supero-temporal side (clockwise for left eyes and anti-clockwise for right eyes, Fig 1A).

#### Macular OCT parameters

For mGCC and mTR imaging, raster scanning over a 9 × 9 mm^2^ area centered on the foveal center was conducted at a scan density of 512 A-scans (horizontal) × 128 B-scans (vertical) [5]. The mGCC thickness was measured between the ILM and the outer boundary of the inner plexiform layer (IPL). The mTR thickness was measured between the ILM and the outer RPE. The RS-3000 OCT provides a macular map based on the glaucoma analysis chart (G Chart). The peripheral concentric areas are divided into eight subfields: supero-temporal, infero-temporal, supero-nasal and infero-nasal for parafoveal (4.5 mm diameter) and perifoveal (9.0 mm diameter) subfields. The 9-mm-diameter circle occasionally overlaps the optic nerve head; therefore, we excluded nasal regions at 180° of the outer ring (Fig 1B). Areas within a 1.5 mm diameter of the foveal center were also excluded.

### Statistical Analyses

All statistical analyses were performed using SPSS statistical software (version 19.0, SPSS Inc., Chicago, IL). The normality of distribution of the study sample was assessed with the Shapiro–Wilk test. Descriptive statistics are presented as means ± standard deviations for normally distributed variables and medians and inter-quartile ranges for non-normally distributed variables. Intrasession reproducibility was evaluated by calculating corresponding intraclass correlation (ICC), coefficient of variation (CV) values and reproducibility coefficient (RC). RC was defined as 2.77 times the intrasession within-subject standard deviation (Sw). Sw was calculated as the square root of the within-subject mean square of error (the unbiased estimator of the component of variance resulting from random error) in a 1-way random effects model [17–19].

## Results

Sixty normal eyes of 30 subjects were included in this study. Seven eyes were excluded because of poor OCT image quality. Four of the seven eyes had an SSI <7, and three eyes had segmentation errors. Fifty-three eyes of 28 subjects were analysed. Patient characteristics are summarised in Table 1.

**Table 1:** Characteristics of the study participants.

Age (years; median (IQR))	33.0 (11)
IOP (mmHg; mean ± SD)	14.7 ± 1.89
Spherical Equivalent (D; mean ± SD)	−3.69 ± 3.43
Axial length (mm; mean ± SD)	25.07± 1.48
MD (dB; mean ± SD)	−0.18 ± 1.04
PSD (dB; mean ± SD)	1.31 ± 0.20

IQR = inter quartile range, D = diopter, IOP = intraocular pressure, MD = mean deviation, PSD = pattern standard deviation

The mean and standard deviation values, and the intrasession ICC, intrasession CV values and the RC values are shown for all tested parameters in Tables 2 and 3, respectively. The results on cpTR thickness and cpRNFL thickness are given in Table 2. Table 2 shows that the intrasession ICC values varied between 0.715 (Sector 11) and 0.926 (Sector 4) for cpRNFL thickness. The intrasession CV was 2.81% for global cpRNFL thickness. The CV values determined for the 12 cpRNFL sectors varied between 5.97% (Sector 5: location, infero-temporal) and 13.98% (Sector 9: location, nasal). For cpTR thickness the intrasession ICC values varied between 0.908 (Sector 8) and 0.986 (global). The intrasession CV was 0.60% for global cpTR thickness. The CV values of the 12 cpTR sectors ranged between 0.86% (Sector 3) and 1.88% (Sector 7). The RC values for the global cpRNFL and cpTR were 9.04 μm and 5.95 μm, respectively. For all cpTR parameters the ICC values were higher, and both the CV and RC values were lower than those for the corresponding cpRNFL parameters.

**Table 2:** Intraobserver reproducibility of cpRNFL thickness (μm) and cpTR thickness (μm).

	cpRNFL				cpTR			
	Mean ± SD	ICC (95% CI)	CV (%)	RC (μm)	Mean ± SD	ICC (95% CI)	CV (%)	RC (μm)
Global	102.53±9.63	0.897 (0.844–0.936)	2.81	9.04	315.93±17.93	0.986 (0.978–0.991)	0.60	5.95
Temporal	77.02±16.27	0.895 (0.841–0.934)	5.96	15.68	309.19±20.45	0.984 (0.975–0.990)	0.70	7.17
Superior	129.08±15.97	0.858 (0.787–0.910)	4.37	17.57	333.09±19.96	0.964 (0.943–0.978)	1.00	10.90
Nasal	72.09±14.14	0.741 (0.629–0.830)	8.82	19.70	284.34±16.28	0.937 (0.899–0.959)	1.30	12.30
Inferior	128.66±16.35	0.887 (0.829–0.929)	4.21	17.84	327.11±22.20	0.959 (0.937–0.975)	1.09	13.89
1	143.85±23.55	0.814 (0.727–0.881)	6.27	31.75	342.74±24.96	0.967 (0.948–0.979)	1.14	12.43
2	90.28±20.44	0.873 (0809–0.920)	6.75	21.18	314.45±23.74	0.973 (0.958–0.983)	1.03	10.87
3	59.28±12.07	0.809 (0.720–0.877)	7.08	15.56	293.19±20.66	0.978 (0.966–0.986)	0.86	8.41
4	78.00±23.29	0.926 (0.886–0.954)	7.19	18.59	306.04±20.69	0.959 (0.937–0.975)	1.15	11.99
5	146.53±23.11	0.817 (0.730–0.882)	5.97	28.96	342.30±22.20	0.948 (0.919–0.968)	1.26	14.55
6	131.62±28.47	0.903 (0.853–0.939)	6.03	28.60	324.26±29.05	0.945 (0.915–0.966)	1.63	19.25
7	101.15±22.21	0.846 (0.771–0.902)	8.00	26.71	298.93±23.77	0.913 (0.86.7–0.946)	1.88	21.06
8	69.40±14.48	0.669 (0.538–0.779)	11.92	24.98	276.57±16.53	0.908 (0.861–0.943)	1.57	14.88
9	58.92±14.73	0.652 (0.517–0.767)	13.98	23.64	269.94±17.01	0.938 (0.905–0.962)	1.32	11.71
10	84.68±21.14	0.760 (0.654–0.844)	10.62	29.50	293.09±19.48	0.914 (0.968–0.946)	1.60	17.20
11	113.34±24.74	0.715 (0.596–0.812)	8.20	36.05	314.45±19.42	0.914 (0.869–0.946)	1.54	16.60
12	122.38±28.43	0.895 (0.841–0.934)	7.23	26.43	326.17±24.83	0.945 (0.915–0.966)	1.58	16.32

CI = confidence interval, cpRNFL = circumpapillary retinal nerve fiber layer, cpTR = circumpapillary total retina, ICC = intraclass correlation, CV = coefficient of variation, RC = reproducibility coefficient

**Table 3:** Intraobserver reproducibility of mGCC thickness (μm) and mTR thickness (μm).

	mGCC				mTR			
	Mean ± SD	ICC(95% CI)	CV (%)	RC (μm)	Mean ± SD	ICC (95% CI)	CV (%)	RC (μm)
Superior	98.08±7.88	0.983 (0.974–0.990)	0.84	2.86	290.17±12.19	0.983 (0.973–0.990)	0.48	4.41
Inferior	98.57±7.64	0.980 (0.968–0.988)	0.98	3.12	285.68±13.33	0.983 (0.973–0.990)	0.43	4.43
1	121.32±8.81	0.979 (0.968–0.987)	0.86	3.45	336.09±14.83	0.985 (0.976–0.991)	0.48	5.04
2	118.57±8.01	0.979 (0.967–0.987)	0.78	3.14	327.55±15.05	0.979 (0.967–0.987)	0.55	5.91
3	108.59±8.62	0.954 (0.929–0.972)	1.32	2.98	320.94±12.87	0.967 (0.948–0.979)	0.63	3.95
4	109.51±7.86	0.949 (0.922–0.969)	1.33	4.95	318.25±13.88	0.964 (0.945–0.978)	0.70	6.55
5	72.28±6.79	0.975 (0.961–0.985)	1.24	4.88	255.74±10.45	0.981 (0.971–0.989)	0.50	7.27
6	74.11±6.99	0.970 (0.954–0.982)	1.36	3.70	251.08±11.59	0.974 (0.960–0.984)	0.63	5.61

CI = confidence interval, mGCC = macular ganglion cell complex, mTR = macular total retina, ICC = intraclass correlation, CV = coefficient of variation, RC = reproducibility coefficient

The results on mTR thickness and mGCC thickness are given in Table 3. Table 3 shows that the intrasession ICC varied between 0.949 (Sector 4) and 0.983 (Superior) for mGCC thickness. The CV values for superior and inferior mGCC thickness were 0.84% and 0.98%, respectively. The RC values for superior and inferior mGCC were 2.86 μm and 3.12 μm, respectively. For mTR thickness the intrasession ICC values varied between 0.964 (Sector 4) and 0.985 (Sector 1). Excellent (generally defined as 0.75–1.00) ICC values were found for all sector mGCC and sector mTR parameters. The CV values for the superior and inferior mTR thickness were 0.48% and 0.43%, respectively. The RC values for superior and inferior mTR were 4.41 μm and 4.43 μm, respectively.

## Discussion

In the current investigation we analysed and compared the short term reproducibility (repeatability) of various corresponding cpRNFL thickness and cp TR thickness; and mGCC thickness and mTR thickness parameters in healthy eyes, respectively. To our best knowledge this is the first OCT study to access the reproducibility of cpTR measurements. The goal of the current investigation was twofold: 1) to identify the most reproducible parameters of the circumpapillary and macular RS-3000 OCT scans for future glaucoma studies, since highly reproducible parameters have a potential to be particularly sensitive to glaucomatous change and progression; 2) to investigate the clinical value of the SLO based image alignment function offered in the RS-3000 OCT without eye tracking.

The background of our investigation was that very recently favourable data on the diagnostic ability of cpTR thickness were published [16]. Simaviliet al. reported that cpTR thickness measured with the Spectralis OCT had similar or better diagnostic accuracy for glaucoma than cpRNFL thickness measured with the same OCT system [16]. For cpTR no segmentation error was observed in the assessment of scans for 156 eyes. In addition, the authors reported that obtaining accurate cpRNFL thickness measurements is more difficult in glaucoma than in healthy eyes due to RNFL thinning and loss of RNFL reflectivity. When RNFL reflectivity is decreased due to moderate and severe glaucoma the segmentation algorithms may fail to correctly detect the posterior RNFL border in all pixels, which may decrease the reproducibility [16]. In contrast, reflectivity of the retinal pigment epithelium/Bruch’s membrane complex, which represents the outer border of cpTR, remains unaltered in glaucoma; and it has been shown that macular outer retinal thickness is in fact not influenced by glaucoma [20–22]. The TR thickness parameters comprise both the inner retinal thickness values (which are influenced by glaucoma) and the outer retinal thickness values (which are not influenced in glaucoma). Thus, clinical value of cpTR and mTR has a potential to exceed that of the extensively used inner retinal thickness parameters (cpRNFL thickness and mGCC thickness) in glaucoma detection and follow-up if cpTR and mTR are more reproducible than the corresponding inner retinal parameters.

Our results showed that ICC for the various cpTR thickness parameters was high up to 0.986 for global cpTR, and all ICC values were higher than those for the corresponding cpRNFL thickness values. In a recent investigation made on healthy eyes the RC of average cpRNFL thickness measured with the Cirrus HD-OCT was 5.12 μm [19]. In the current investigation, the RC for global cpRNFL was 9.04 μm, but for global cpTR it was 5.95 μm. RCs for all cpTR thickness parameters were lower than for the corresponding cpRNFL parameters. The CV for global cpTR was only 0.60%. Our CV values for the cpTR parameters were even lower than those reported by others for cpRNFL parameters measured with other OCT systems [23–27]. In our study the CV values for the various cpRNFL thickness parameters were 4 to 10 times higher than for the corresponding cpTR parameters.

For the macular area the differences in reproducibility were less extensive than for the circumpapillary area. The ICC and CV values were similarly favourable for the corresponding mGCC and mTR thickness measurements. No mTR parameter had a CV value higher than 0.70%, and the CV values for the superior and inferior mTR were only 0.48% and 0.43%, respectively. Our results confirm the recently published reproducibility data by Giani *et al*., who reported a high rate of reproducibility for macular retina thickness measurements with the RS-3000 [14]. In a recently published study RC determined with the Cirrus-HD OCT for macular ganglion cell-inner plexiform layer thickness of healthy eyes was 1.88 μm [28]. In the current investigation it was approximately 3.0 μm for mGCC thickness and approximately 4.4 μm for mTR thickness. Since mTR thickness is higher than mGCC thickness the higher RC value for mTR was expected.

The results of our investigation suggest that the various TR thickness parameters of the RS-3000 OCT are in fact highly reproducible, and therefore may potentially be investigated for glaucoma diagnostic accuracy and accuracy in detection of glaucomatous progression. At the same time our results and the previously published results obtained with another OCT system [11] suggest that the benefits found in the current study for the TR thickness parameters may represent a general feature, and therefore their evaluation in various OCT systems is proposed.

**Fig 2:**
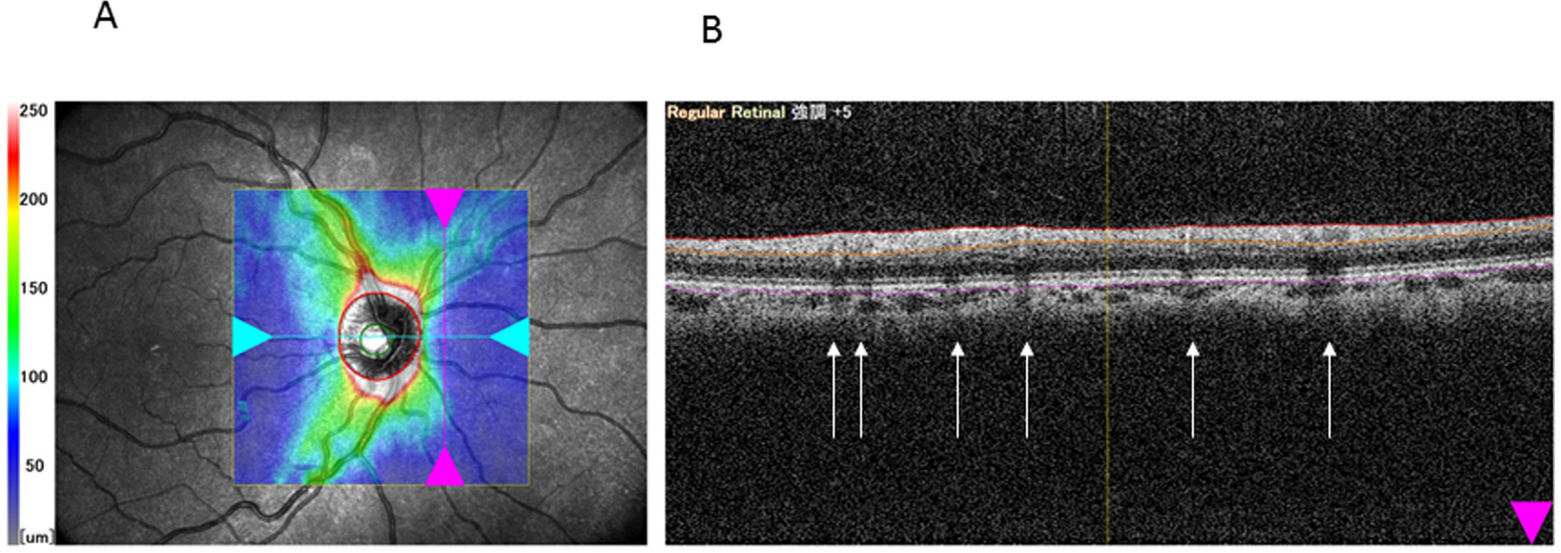
An optical coherence tomography image of the peripapillary retina made with the RS-3000 instrument, and its position on the fundus (A). The signals arriving from retinal layers beneath the main retinal vessels are attenuated due to light absorption by the overlying vessels (B). The locations of this shadow effect are indicated with arrows.

Our study made it also possible to evaluate the SLO based image alignment function of the RS-3000 OCT. Though the repeatability figures for the various TR thickness parameters were highly satisfactory, the corresponding figures for cpRNFL thickness were relatively low, even when compared to published results obtained with other OCT systems [23, 25, 29]. Since the same images were used to calculate the CV and ICC values for all parameters including those which were highly reproducible, we think that the image alignment function used by us is clinically useful, and cannot be considered as the reason of the lower repeatability of the cpRNFL thickness parameters compared to cpTR thickness. It has been suggested that retinal vessels in the RNFL may cause measurement noise and artefacts in B scan images, which affect the RNFL thickness measurements, particularly in the circumpapillary area where the vessels are large [30]. This effect, however, is present in all OCT systems. Thus our less favourable repeatability figures for cpRNFL thickness cannot be explained with the general aspects of the vessel related noise. However we used an SLO based image alignment function which was not used in the repeatability studies published for other OCT systems. Though the details of the image alignment software are not available for the users, one may speculate that the vessel based alignment had a negative influence on the vessel induced measurement noise (Fig 2).

Our study has limitations. The sample size was relatively small, all eyes were non-glaucomatous healthy eyes, and all participants were Japanese. Therefore no direct conclusion from our results can be made for glaucoma patients and other ethnic groups. In the current pilot study we did not include a glaucoma group with a wide range of disease severity, since we wished to clarify those circumpapillary and macular parameters which seem to be most useful for our future glaucoma diagnostic studies.

In conclusion, in this repeatability study made on healthy Japanese eyes all global, quadrant and sector cpTR parameters showed excellent intrasession reproducibility, which was better than that found for the corresponding cpRNFL thickness parameters. As far as we know this is the first evaluation of repeatability of cpTR. A favourable repeatability was also found for the mTR parameters. Our results suggest that the cpTR and mTR parameters are of potential clinical usefulness both for glaucoma diagnostics and detection of glaucomatous structural progression. To evaluate accuracy of these parameters to detect glaucoma and progression of glaucoma further clinical studies are needed.
